# Do hospital workers experience a higher risk of respiratory symptoms and loss of lung function?

**DOI:** 10.1186/s12890-022-02098-5

**Published:** 2022-08-08

**Authors:** Behzad Heibati, Maritta S. Jaakkola, Taina K. Lajunen, Alan Ducatman, Rahmat Veysi, Ali Karimi, Jouni J. K. Jaakkola

**Affiliations:** 1 Faculty of Medicine, Center for Environmental and Respiratory Health Research, University of Oulu, Aapistie 5B, P.O. Box 5000, 90014 Oulu, Finland; 2 Faculty of Medicine, Biocenter Oulu, University of Oulu, P.O. Box 5000, 90014 Oulu, Finland; 3 Medical Research Center Oulu, Oulu University Hospital, University of Oulu, P.O. Box 8000, 90014 Oulu, Finland; 4 West Virginia University School of Public Health, Morgantown, WV USA; 5 Department of Occupational Health Engineering, School of Public Health, Shiraz University of Medical Sciences, Shiraz, Iran; 6 Department of Occupational Health, School of Public Health, Tehran University of Medical Sciences, Tehran, Iran; 7 Finnish Meteorological Institute, P.O. Box 503, 00101 Helsinki, Finland

**Keywords:** Occupational exposures, Pulmonary function, Respiratory symptoms, Hospital workers

## Abstract

**Background:**

Hospital work environment contains various biological and chemical exposures that can affect indoor air quality and have impact on respiratory health of the staff. The objective of this study was to investigate potential effects of occupational exposures on the risk of respiratory symptoms and lung function in hospital work, and to evaluate potential interaction between smoking and occupational exposures.

**Methods:**

We conducted a cross-sectional study of 228 staff members in a hospital and 228 employees of an office building as the reference group in Shiraz, Iran. All subjects completed a standardized ATS respiratory questionnaire and performed a spirometry test.

**Results:**

In Poisson regression, the adjusted prevalence ratios (aPR) among the hospital staff were elevated for cough (aPR 1.90, 95% CI 1.15, 3.16), phlegm production (aPR 3.21, 95% CI 1.63, 6.32), productive cough (aPR 2.83, 95% CI 1.48, 5.43), wheezing (aPR 3.18, 95% CI 1.04, 9.66), shortness of breath (aPR 1.40, 95% CI 0.93, 2.12), and chest tightness (aPR 1.73, 95% CI 0.73, 4.12). Particularly laboratory personnel experienced increased risks of most symptoms. In linear regression adjusting for confounding, there were no significant differences in lung function between the hospital and office workers. There was an indication of synergism between hospital exposures and current smoking on FEV1/FVC% (interaction term β = − 5.37, 95% CI − 10.27, − 0.47).

**Conclusions:**

We present significant relations between hospital work, especially in laboratories, and increased risks of respiratory symptoms. Smoking appears to enhance these effects considerably. Our findings suggest that policymakers should implement evidence-based measures to prevent these occupational exposures.

**Supplementary Information:**

The online version contains supplementary material available at 10.1186/s12890-022-02098-5.

## Background

Increased risks of respiratory symptoms have been reported in several occupational groups, including bakers and cooks, construction workers, hairdressers and cosmetologists, farmers, fisherman, workers in chemical, plastic and rubber industries, laboratory technicians and hospital workers, florists and greenhouse workers, and cleaners [1–5]. Cigarette smoking is a risk factor for several respiratory symptoms and diseases [6, 7]. A study based on the US Behavioral Risk Factor Surveillance System worker data reported that healthcare workers were more likely to report recent respiratory symptoms than workers in general [8]. A more detailed survey of healthcare professionals from four groups of Texas health professionals, licensed as of 2003, including physicians, nurses, occupational therapists, and respiratory therapists, found that those working with instrument cleaning and administration of aerosolized medications showed a higher occurrence of adverse respiratory outcomes. In contrast, the risk of symptoms and diseases related to the use of latex gloves decreased since the year 2000 [4]. A recent survey of more than 2000 New York healthcare workers showed that use of alcohol, bleach, and other disinfectants was associated with reported asthma symptoms [9, 10]. No previous study has addressed potential interaction between smoking and occupational exposures containing disinfectant products.

The objective of this study was to investigate potential effects of occupational exposures among hospital workers on the risk of respiratory symptoms and lung function level in Shiraz, Iran, and to evaluate potential interaction between occupational exposures and smoking.

## Methods

### Study design and study population

This was a cross-sectional study conducted in 2015. The study population comprised of 228 hospital workers of a large teaching hospital in Shiraz, Iran, and a reference group of 228 office workers recruited from the same area nearby the hospital. The reference group workers were in managerial, administrative, or clerical jobs, and they visited the hospital only occasionally. To be included in this study the participants had to have at least 1 year of work experience in the current job. The exclusion criteria included any history of previous respiratory or heart diseases, any history of asthma, chest or heart surgery, recent eye or ear surgery, heart attack, stroke, bloody phlegm (i.e. haemoptysis), systolic blood pressure above 180 mmHg, recent severe common cold, or previous exposure to toxic pollutants in other occupations. The sample size was based on a comparison of the prevalences of respiratory symptoms between the exposed and the reference groups (beta = 90%, alpha = 0.05).

### Data collection

Both the exposed and reference groups answered a set of standardized questions modified from the American Thoracic Society’s (ATS) Respiratory questionnaire [11], which has been validated in multiple populations around the world [12–14].

This questionnaire inquired about respiratory symptoms, including current cough, productive cough, phlegm production, wheezing, shortness of breath, and chest tightness. It contained questions on age, gender, weight, height, working history, current job title, current workplace, past occupations, history of previous diseases, and some additional demographic characteristics. In addition, the participants were asked to fill in a checklist of the work environment characteristics, including the dimensions of the workspace, number of people working in the same area, ventilation system of the workplace, and availability of heating and cooling systems. The questionnaire inquired also about job tasks and their duration, chemical products used, availability and application of chemical safety guidelines, use of personal protective equipment, and training in occupational health issues. These questions were modified to suit the Iranian environment, then translated to Farsi and then back-translated to English by a different person. The back-translated questionnaire was then compared to the original version, and corrections were made in the translated questionnaire.

### Exposure assessment

We assessed occupational exposure on the basis of the study subject’s job category. First, all the healthcare workers constituted the exposed group and office workers formed the reference group. Second, we formed 6 subcategories with different types of exposures: nurses, laboratory workers, nurses’ aides, cleaners, surgical technicians, and others. Unfortunately, the study size was not large enough to address effects of individual exposures.

### Outcome assessment

The main outcomes of interest were the occurrence of six respiratory symptoms, including (1) cough, (2) mucus production, (3) cough accompanied by mucus, (4) wheezing, (5) shortness of breath, and (6) chest tightness, and three lung function parameters, including (1) forced vital capacity (FVC), (2) forced expiratory volume in one second (FEV1), and (3) FEV1/FVC ratio. Both absolute values and percentage predicted values of the lung function parameters were used as outcomes. Forced expiratory maneuvers were performed according to the ATS/ERS guidelines using a Spiroanalyzer ST-150 spirometer (Fukuda Sangyo Inc, Japan). The equipment was calibrated every four hours according to the manufacturer's recommendations. Predicted values were derived from the GLI reference equation [15]. Spirometry was conducted by the same trained, experienced technician for both groups at their workplaces. The study was approved by the ethics committee of the Shiraz University of Medical Sciences. All participants signed informed consent before participation.

### Statistical methods

We compared the risk of respiratory symptoms and the levels of lung function parameters between hospital workers (the exposed group) and office workers (the reference group). Prevalence ratio (PR) was used as the measure of effect. We adjusted the effect estimates for potential confounding in Poisson regression analyses, producing prevalence ratios (PR) with corresponding 95% confidence intervals (CI). Poisson regression models were fitted applying SAS procedure GENMOD, with logarithmic link function [16]. Multiple linear regression models were applied to estimate the effects of occupational exposures on the lung function parameters. The effect estimates were adjusted for age, height, marital status, education, sex (model 1), then additionally for smoking (never, ex, and current), and use of waterpipe (never, ex, and current) (model 2).

We assessed the excess relative risks (ERR) for the joint effects of occupational exposures and smoking status on the risk of the five studied symptoms. The relative risk due to interaction (RERI) was quantified on an additive scale by calculating the risk that is more than expected based on summing the independent effects of these factors. This can be expressed in terms of ERRs as follows:

$${{RERI}}\, = \,{{ERR }}\left( {{{AB}}} \right) - {{ERR }}\left( {{A}} \right) - {{ERR }}\left( {{B}} \right)$$

We estimated the 95% CI for RERI using the method of variance estimates recovery [17]. For RERI, the null value corresponds to a statistical significance level *p* = 0.05. Only the estimates for which sufficient information for calculation of ERRs and estimation of RERIs was available are given in results. Data was analyzed using SAS statistical package v.9.4 (SAS Institute Inc., Cary, NC, USA).

## Results

### Characteristics of the study population

The characteristics of the exposed and reference groups are presented in Table 1. Most of the characteristics were similar between the two groups. However, hospital staff had longer work experience than the reference group, and a shorter duration of education. Hospital staff reported current smoking more frequently, although there was no significant difference with respect to the lifetime smoking. In addition, use of a face mask was reported by 67.1% of the exposed participants overall, and was most frequent among operating room workers (100%), followed by cleaners (90.9%), nurses’ aides (75.7%), nurses (62.8%), laboratory workers (50.0%), and others (36.4%). Availability of a local exhaust system was reported by 9.7% of the exposed group.

**Table 1:** Characteristics of the study population of 228 healthcare workers (HCWs) and 228 reference group (office workers)

Characteristic	Healthcare workers (exposed group) (n = 228)	Office workers (reference group) (n = 228)	*p*-value
Age (year), mean ± SD	36.3 ± 8.25	35.5 ± 6.65	0.39^a^
Height (cm), mean ± SD	165.8 ± 9.0	165.5 ± 8.60	0.80^a^
Weight (kg), mean ± SD	69.4 ± 14.40	69.6 ± 12.70	0.51^a^
BMI (kg/m^2^), mean ± SD	25.2 ± 4.70	25.3 ± 3.60	0.29
Work history (years), mean ± SD	12.0 ± 7.46	8.9 ± 5.64	< 0.0001^a^
Average working hours/week, mean ± SD	51.7 ± 13.08	45.0 ± 10.11	< 0.0001^a^
*Any smoking status, n (%)*			0.42^b^
Current	13 (5.7)	6 (2.7)	
Past	4 (1.8)	11 (4.8)	
Never	211 (92.5)	211 (92.5)	
*Waterpipe status, n (%)*			0.58^b^
Current	4 (1.7)	3 (1.3)	
Past	7 (3.1)	5 (2.2)	
Never	216 (95.2)	217 (96.5)	
*Sex, n (%)*			0.84^b^
Male	79 (34.6)	77 (33.8)	
Female	149 (65.4)	151 (66.2)	
*Marital status, n (%)*			0.25^b^
Single	61 (26.8)	72 (31.6)	
Married	167 (73.2)	156 (68.4)	
*Education status, n (%)*			< 0.0001^b^
Grade School or Junior High	33 (14.5)	6 (2.6)	
High School	78 (34.2)	72 (31.6)	
Post High School-Technical School	102 (44.7)	99 (43.4)	
College/university	15 (6.6)	51 (22.4)	
*Job exposure category, n (%)*			< 0.0001^b^
Office	0 (0)	228 (100.0)	
Nurses	86 (37.7)	0 (0)	
Laboratory workers	20 (8.8)	0 (0)	
Nurses’ aides	37 (16.2)	0 (0)	
Cleaners	33 (14.5)	0 (0)	
Surgical technicians	19 (8.3)	0 (0)	
Others	33 (14.5)	0 (0)	

Information on waterpipe status is missing for one person in the hospital staff group, and for three persons in the reference group

^a^Mann–Whitney U test

^b^χ2 test

### Prevalence of lung function impairment among the exposed and control groups

Table 2 shows the prevalence of lung function impairment in the four categories of the exposed group and the reference group. The overall prevalence of lung function impairment was 22.0% (n = 50) in the exposed group and 17.5% (n = 40) in the reference group. Within the exposed group, 16.7% showed a restrictive pattern while 5.3% showed obstruction. In the reference group the majority of those with lung function impairment had restriction 29 (12.7%), followed by obstruction 11 (4.8%). Obstruction in combination with restrictive impairment was not observed in either group.

**Table 2:** Prevalence of lung function deficits

Exposure category	Obstruction^a^	Restriction^b^	Combination^c^	Normal^d^
Reference group (*n* = 228)	11 (4.8%)	29 (12.7%)	NA	188 (82.5%)
Exposed group (*n* = 228)	12 (5.3%)	38 (16.7%)	NA	178 (78.0%)
Nurses (*n* = 86)	6 (7.0%)	16 (18.6%)	NA	64 (74.4%)
Laboratory workers (*n* = 20)	1 (5.0%)	2 (10.0%)	NA	17 (85.0%)
Nurses’ aides (*n* = 37)	0 (0%)	8 (21.6%)	NA	29 (78.4%)
Cleaners (*n* = 33)	3 (9.0%)	5 (15.2%)	NA	25 (75.8%)
Surgical technicians (*n* = 19)	0 (0%)	5 (26.3%)	NA	14 (73.7%)
Others (*n* = 33)	2 (6.0%)	2 (6.0%)	NA	29 (88.0%)

^a^FVC normal (≥ 80%), FEV1 reduced (< 80%), FEV1/FVC reduced (< 0.7)

^b^FVC reduced (< 80%), FEV1 normal or reduced (≥ 80% OR < 80%), FEV1/FVC normal or increased (≥ 0.7)

^c^FVC reduced (< 80%), FEV1 reduced (< 80%), FEV1/FVC reduced (< 0.7)

^d^FVC normal (≥ 80%), FEV1 normal (≥ 80%), FEV1/FVC normal (≥ 0.7)

### Effects of exposure on respiratory symptoms

The prevalences of respiratory symptoms among the hospital and the reference groups are reported in Table 3. The prevalences of all symptoms were higher in the hospital workers compared to the office workers. The results showed that even after adjusting for potential confounders, significantly higher PRs were found in the exposed hospital staff group with adjusted PRs for cough 1.90 (95% CI 1.15, 3.16), phlegm production 3.21 (95% CI 1.63, 6.32), productive cough 2.83 (95% CI 1.48, 5.43), and wheezing 3.18 (95% CI 1.04, 9.66) (Table 3).

**Table 3:** Prevalence ratio (PR) of respiratory symptoms among HCWs compared to the reference group of office workers

Symptom	Reference group (*n* = 228) *n* (%)	Exposed group (*n* = 228) *n* (%)	Unadjusted model PR (95% CI)	Adjusted model 1^a^ PR (95% CI)	Adjusted model 2^b^ PR (95% CI)
Cough	25 (11.0)	54 (23.7)	**2.16 (1.34, 3.47)**	**1.96 (1.19, 3.24)**	**1.90 (1.15, 3.16)**
Phlegm production	12 (5.3)	40 (17.5)	**3.33 (1.74, 6.35)**	**3.10 (1.59, 6.02)**	**3.21 (1.63, 6.32)**
Productive cough	15 (6.6)	37 (16.2)	**2.47 (1.35, 4.49)**	**2.66 (1.41, 4.99)**	**2.83 (1.48, 5.43)**
Wheezing	4 (1.8)	17 (7.5)	**4.25 (1.43, 12.63)**	**3.38 (1.12, 10.22)**	**3.18 (1.04, 9.66)**
Shortness of breath	43 (18.9)	71 (31.1)	**1.65 (1.13, 2.41)**	1.42 (0.95, 2.14)	1.40 (0.93, 2.12)
Chest tightness	10 (4.4)	18 (7.9)	1.80 (0.83, 3.89)	1.79 (0.77, 4.18)	1.73 (0.73, 4.12)
*Symptom*					
Exposure category	Cough				
Reference group (*n* = 228)	25 (11.0)		Ref	Ref	Ref
Nurses (*n* = 86)	16 (18.6)		1.70 (0.90, 3.17)	1.81 (0.90, 3.63)	1.74 (0.87, 3.49)
Laboratory workers (*n* = 20)	9 (45.0)		**4.10 (1.91, 8.79)**	**4.08 (1.85, 9.00)**	**4.29 (1.92, 9.56)**
Nurses’ aides (*n* = 37)	10 (27.0)		**2.46 (1.18, 5.13)**	1.78 (0.79, 4.03)	1.68 (0.73, 3.85)
Cleaners (*n* = 33)	8 (24.2)		2.21 (0.99, 4.90)	1.27 (0.46, 3.45)	1.32 (0.48, 3.62)
Surgical technicians (*n* = 19)	2 (10.5)		0.96 (0.22, 4.05)	1.02 (0.24, 4.35)	0.94 (0.22, 4.04)
Others (*n* = 33)	9 (27.3)		**2.49 (1.16, 5.32)**	2.19 (0.96, 4.98)	2.07 (0.88, 4.86)
*Phlegm production*					
Reference group (*n* = 228)	12 (5.3)		Ref	Ref	Ref
Nurses (*n* = 86)	15 (17.4)		**3.31 (1.55, 7.07)**	**4.20 (1.74, 10.10)**	**4.02 (1.66, 9.72)**
Laboratory workers (*n* = 20)	3 (15.0)		2.85 (0.80, 10.09)	3.21 (0.88, 11.67)	**3.81 (1.03, 14.13)**
Nurses’ aides (*n* = 37)	9 (24.3)		**4.62 (1.94, 10.96)**	**2.93 (1.15, 7.44)**	**3.13 (1.20, 8.16)**
Cleaners (*n* = 33)	4 (12.1)		2.30 (0.74, 7.14)	1.40 (0.37, 5.23)	1.59 (0.43, 5.82)
Surgical technicians (*n* = 19)	1 (5.3)		1.00 (0.13, 7.69)	0.99 (0.12, 7.66)	1.02 (0.13, 8.04)
Others (*n* = 33)	8 (24.2)		**4.60 (1.88, 11.26)**	**3.58 (1.35, 9.43)**	**3.95 (1.45, 10.73)**
*Productive Cough*					
Reference group (*n* = 228)	15 (6.6)		Ref	Ref	Ref
Nurses (*n* = 86)	10 (11.6)		1.76 (0.79, 3.93)	1.90 (0.79, 4.60)	1.91 (0.78, 4.69)
Laboratory workers (*n* = 20)	7 (35.0)		**5.32 (2.16, 13.04)**	**6.37 (2.50, 16.22)**	**6.72 (2.58, 17.46)**
Nurses’ aides (*n* = 37)	9 (24.3)		**3.69 (1.62, 8.45)**	**3.47 (1.33, 9.09)**	**4.09 (1.51, 11.09)**
Cleaners (*n* = 33)	5 (15.2)		2.30 (0.83, 6.33)	2.22 (0.62, 7.87)	2.53 (0.69, 9.18)
Surgical technicians (*n* = 19)	1 (5.3)		0.80 (0.10, 6.05)	0.88 (0.11, 6.76)	0.94 (0.12, 7.24)
Others (*n* = 33)	5 (15.2)		2.30 (0.83, 6.33)	2.61 (0.90, 7.51)	2.85 (0.98, 8.31)
*Wheezing*					
Reference group (*n* = 228)	4 (1.8)		Ref	Ref	Ref
Nurses (*n* = 86)	2 (2.3)		1.32 (0.24, 7.23)	1.64 (0.25,10.56)	1.49 (0.24, 9.06)
Laboratory workers (*n* = 20)	4 (20.0)		**11.40 (2.85, 45.58)**	**7.51 (1.79, 31.44)**	**10.41 (2.35, 45.97)**
Nurses’ aides (*n* = 37)	5 (13.5)		**7.70 (2.06, 28.68)**	**4.11 (1.02, 16.51)**	3.64 (0.85, 15.59)
Cleaners (*n* = 33)	4 (12.1)		**6.90 (1.72, 27.62)**	4.82 (0.95, 24.25)	**5.07 (1.0, 25.60)**
Surgical technicians (*n* = 19)	1 (5.3)		3.0 (0.33, 26.84)	2.47 (0.27, 22.39)	1.85 (0.19, 18.08)
Others (*n* = 33)	1 (3.0)		1.72 (0.19, 15.45)	1.40 (0.15, 13.20)	1.15 (0.12, 11.06)
*Shortness of breath*					
Reference group (*n* = 228)	43 (18.9)		Ref	Ref	Ref
Nurses (*n* = 86)	26 (30.2)		1.60 (0.98, 2.60)	1.58 (0.92, 2.71)	1.49 (0.56, 3.90)
Laboratory workers (*n* = 20)	7 (35.0)		1.85 (0.83, 4.12)	1.96 (0.86, 4.45)	2.01 (0.88, 4.63)
Nurses’ aides (*n* = 37)	13 (35.1)		**1.86 (1.0, 3.46)**	1.35 (0.66, 2.74)	1.32 (0.64, 2.72)
Cleaners (*n* = 33)	13 (39.4)		**2.08 (1.12, 3.88)**	0.95 (0.40, 2.22)	0.95 (0.39, 2.30)
Surgical technicians (*n* = 19)	5 (26.3)		1.39 (0.55, 3.52)	1.55 (0.61, 3.95)	1.55 (0.60, 3.98)
Others (*n* = 33)	7 (21.2)		1.12 (0.51, 2.50)	0.99 (0.42, 2.32)	1.00 (0.41, 2.41)
*Chest tightness*					
Reference group (*n* = 228)	10 (4.4)		Ref	Ref	Ref
Nurses (*n* = 86)	4 (4.7)		1.06 (0.33, 3.381)	1.36 (0.37, 4.92)	1.36 (0.37, 4.93)
Laboratory workers (*n* = 20)	4 (20.0)		**4.56 (1.43, 14.53)**	**4.44 (1.30, 15.16)**	**4.38 (1.27, 15.05)**
Nurses’ aides (*n* = 37)	4 (10.8)		2.46 (0.77, 7.85)	1.66 (0.41, 6.68)	1.54 (0.37, 6.28)
Cleaners (*n* = 33)	3 (9.1)		2.07 (0.57, 7.53)	1.02 (0.18, 5.85)	1.03 (0.17, 6.30)
Surgical technicians (*n* = 19)	1 (5.3)		1.20 (0.15, 9.37)	1.61 (0.20, 12.76)	1.40 (0.17, 11.25)
Others (*n* = 33)	2 (6.1)		1.38 (0.30, 6.30)	1.42 (0.28, 7.07)	1.19 (0.22, 6.46)

Bolded PR's are statistically significant at *p* < 0.05

^a^Adjusted for age, marital status, education, sex, and BMI

^b^Adjusted for age, marital status, education, sex, BMI, smoking (never, ex, and current), and waterpipe (never, ex, and current)

Among the hospital workers, those working in laboratories showed the highest occurrence of respiratory symptoms. In Poisson regression models adjusting for confounding, all the effect estimates were significantly increased, including cough (aPR = 4.29, 95% CI 1.92, 9.56), phlegm production (aPR = 3.81, 95% CI 1.03, 14.13), productive cough (aPR = 6.72, 95% CI 2.58, 17.46), wheezing (aPR = 10.41, 95% CI 2.35, 45.97), and chest tightness (aPR = 4.38, 95% CI 1.27, 15.05). Furthermore, the risk of phlegm production (aPR = 3.13, 95% CI 1.20, 8.16) and productive cough (aPR = 4.09, 95% CI 1.51, 11.09) were significantly higher among nurses’ aides compared to the reference group. The PR for reported phlegm production was increased in nurses even in the fully adjusted model (aPR = 4.02, 95% CI 1.66, 9.72) compared to the office staff. In contrast, surgical technicians were not found to be at increased risk of experiencing more symptoms than the reference group.

### Effects of exposure on lung function

Additional file 1: Table S1 presents the estimated differences in lung function parameters between the exposed and the reference groups. The negative values represent adverse effects. None of the effect estimates for hospital workers analyzed collectively were statistically significant, although the direction of effect was negative (i.e. worse lung function in the exposed group) for most estimates. However, Additional file 1: Table S1 also presents comparisons between subgroups of the exposure and the reference groups. In the unadjusted models, the nurse subgroup showed a statistically significant decrease in FEV1 (unadjusted difference = − 0.20 L, 95% CI − 0.36, − 0.03) and FVC (unadjusted difference = − 0.25 L, 95% CI − 0.45, − 0.06) compared to the reference group, although the differences were not significant after adjusting for confounding.

### Synergistic effect of occupational exposures and smoking

Tables 4 and 5 present the results of studying potential interaction between occupational exposures and smoking (both former and current smoking) on the risk of respiratory symptoms and lung function levels. Table 4 shows estimates for the independent and joint effects of current smoking and occupational exposures and the corresponding excess relative risks (ERRs) and relative risks due to interaction (RERIs). The observations of RERI should be interpreted with caution, because an analysis of joint effects would ideally be derived from a larger study population.

**Table 4:** Interaction between smoking and occupational exposure among health care workers on the risk of symptoms

Exposure category	Symptom n (%)	Unadjusted model PR (95% CI)	Adjusted Model^a^ PR (95% CI)	ERR (95% CI)	RERI (95% CI)
*Cough*					
Reference, *n* = 211	23 (10.9)	Ref	Ref	Ref	
Exposure only, *n* = 211	47 (22.3)	**2.04 (1.24, 3.36)**	**1.86 (1.10, 3.14)**	0.86 (0.10, 2.14)	
Current smoking only, *n* = 6	1 (16.7)	1.53 (0.20, 11.32)	1.61 (0.21, 12.15)	0.61 (− 0.79, 11.15)	
Exposure and current smoking, *n* = 13	6 (46.2)	**4.23 (1.72, 10.40)**	**4.37 (1.53, 12.52)**	**3.37 (0.53, 11.52)**	1.90 (− 8.81, 9.76)
Ex-smoking only, *n* = 11	1 (9.1)	0.83 (0.11, 6.17)	0.83 (0.10, 6.65)	− 0.17 (− 0.90, 5.65)	
Exposure and ex-smoking, *n* = 4	1 (25.0)	2.29 (0.31, 16.98)	2.46 (0.31, 19.57)	1.46 (− 0.69, 18.57)	0.77 (− 5.56, 17.74)
Reference, *n* = 211	23 (10.9)	Ref	Ref	Ref	
Exposure only, *n* = 211	47 (22.3)	**2.04 (1.24, 3.36)**	**1.86 (1.10, 3.14)**	**0.86 (0.10, 2.14)**	
Current or ex-smoking only *n* = 17	2 (11.8)	1.08 (0.25, 4.58)	1.10 (0.24, 4.98)	0.10 (− 0.76, 3.98)	
Exposure and current or ex-smoking, *n* = 17	7 (41.2)	**3.78 (1.62, 8.80)**	**3.94 (1.48, 10.51)**	**2.94 (0.48, 9.51)**	1.98 (− 2.24, 8.15)
*Phlegm production*					
Reference, *n* = 211	9 (4.3)	Ref	Ref	Ref	
Exposure only, *n* = 211	34 (16.1)	**3.78 (1.81, 7.88)**	**3.60 (1.69, 7.65)**	**2.60 (0.69, 6.65)**	
Current smoking only, *n* = 6	1 (16.7)	3.91 (0.49, 30.84)	3.88 (0.48, 31.61)	2.88 (− 0.52, 30.61)	
Exposure and current smoking, *n* = 13	5 (38.5)	**9.02 (3.02, 26.90)**	**10.03 (2.87, 34.99)**	**9.03 (1.87, 33.99)**	3.55 (− 24.40, 27.17)
Ex-smoking only, *n* = 11	2 (18.2)	4.26 (0.92, 19.73)	4.73 (0.93, 24.09)	3.73 (− 0.07, 23.09)	
Exposure and ex-smoking, *n* = 4	1 (25.0)	5.86 (0.74, 46.26)	4.58 (0.54, 38.93)	3.58 (− 0.46, 37.93)	− 2.75 (− 23.46, 30.7)
Reference, *n* = 211	9 (4.3)	Ref	Ref	Ref	
Exposure only, *n* = 211	34 (16.1)	**3.78 (1.81, 7.88)**	**3.60 (1.69, 7.65)**	**2.60 (0.69, 6.65)**	
Current or ex-smoking only, *n* = 17	3 (17.6)	**4.14 (1.12, 15.28)**	**4.36 (1.10, 17.34)**	**3.36 (0.10, 16.34)**	
Exposure and Current or ex-smoking, *n* = 17	6 (35.3)	**8.27 (2.94, 23.25)**	**8.18 (2.56, 26.12)**	**7.18 (1.56, 25.12)**	1.25 (− 12.06, 17.31)
*Productive cough*					
Reference, *n* = 211	14 (6.6)	Ref	Ref	Ref	
Exposure only, *n* = 211	35 (16.6)	**2.50 (1.34, 4.65)**	**2.73 (1.43, 5.24)**	**1.73 (0.43, 4.24)**	
Current smoking only, *n* = 6	0	NA	NA	NA	
Exposure and current smoking, *n* = 13	2 (15.4)	2.32 (0.53, 10.20)	3.62 (0.72, 18.29)	2.62 (− 0.28, 17.29)	NA
Ex-smoking only, *n* = 11	1 (9.1)	1.37 (0.18, 10.42)	1.99 (0.24, 16.72)	0.99 (− 0.76, 15.72)	
Exposure and ex-smoking, *n* = 4	0	NA	NA	NA	NA
Reference, *n* = 211	14 (6.6)	Ref	Ref	Ref	
Exposure only, *n* = 211	35 (16.6)	**2.50 (1.34, 4.65)**	**2.73 (1.43, 5.24)**	**1.73 (0.43, 4.24)**	
Current or ex-smoking only, *n* = 17	1 (5.9)	0.89 (0.12, 6.74)	1.09 (0.14, 8.79)	0.09 (− 0.86, 7.79)	
Exposure and Current or ex-smoking, *n* = 17	2 (11.8)	1.77 (0.40, 7.80)	2.32 (0.47, 11.36)	1.32 (− 0.53, 10.36)	− 0.50 (− 8.82, 8.17)
*Wheezing*					
Reference, *n* = 211	3 (1.4)	Ref	Ref	Ref	
Exposure only, *n* = 211	12 (5.7)	**4.00 (1.13, 14.17)**	3.18 (0.89, 11.43)	2.18 (− 0.11, 10.43)	
Current smoking only, *n* = 6	0	NA	NA	NA	
Exposure and current smoking, *n* = 13	4 (30.8)	**21.64 (4.84, 96.69)**	**15.81 (2.73, 91.48)**	**14.81 (1.73, 90.48)**	NA
Ex-smoking only, *n* = 11	1 (9.1)	6.39 (0.66, 61.47)	5.68 (0.48, 66.90)	4.68 (− 0.52, 6.90)	
Exposure and ex-smoking, *n* = 4	1 (25.0)	**17.58 (1.83, 169.04)**	**12.71 (1.12, 144.11)**	**11.71 (0.12, 143.11)**	4.85 (− 57.21, 133.87)
Reference, *n* = 211	3 (1.4)	Ref	Ref	Ref	
Exposure only, *n* = 211	12 (5.7)	**4.00 (1.13, 14.17)**	3.18 (0.89, 11.43)	2.18 (− 0.11, 10.43)	
Current or ex-smoking only, *n* = 17	1 (5.9)	4.14 (0.43, 39.77)	3.32 (0.31, 35.56)	2.32 (− 0.69, 34.56)	
Exposure and Current or ex-smoking, *n* = 17	5 (29.4)	**20.69 (4.94, 86.56)**	**14.13 (2.71, 73.56)**	**13.13 (1.71, 72.56)**	8.63 (− 23.07, 65.40)
*Shortness of breath*					
Reference, *n* = 211	37 (17.5)	Ref	Ref	Ref	
Exposure only, *n* = 211	68 (32.2)	**1.84 (1.23, 2.74)**	**1.59 (1.03, 2.44)**	**0.59 (0.03, 1.44)**	
Current smoking only, *n* = 6	3 (50.0)	2.85 (0.88, 9.25)	**3.83 (1.14, 12.86)**	**2.83 (0.14, 11.86)**	
Exposure and current smoking, *n* = 13	3 (23.1)	1.32 (0.40, 4.27)	1.68 (0.46, 6.08)	0.68 (− 0.54, 5.08)	− 2.75 (− 11.87, 2.09)
Ex-smoking only, *n* = 11	3 (27.3)	1.56 (0.48, 5.04)	1.79 (0.49, 6.54)	0.79 (− 0.51, 5.54)	
Exposure and ex-smoking, *n* = 4	0	NA	NA	NA	NA
Reference, *n* = 211	37 (17.5)	Ref	Ref	Ref	
Exposure only, *n* = 211	68 (32.2)	**1.84 (1.23, 2.74)**	**1.59 (1.03, 2.44)**	**0.59 (0.03, 1.44)**	
Current or ex-smoking only, *n* = 17	6 (35.3)	2.01 (0.85, 4.77)	2.52 (0.96, 6.58)	1.52 (− 0.04, 5.58)	
Exposure and Current or ex-smoking, *n* = 17	3 (17.6)	1.00 (0.31, 3.26)	1.37 (0.39, 4.87)	0.37 (− 0.61, 3.87)	− 1.75 (− 5.98, 1.71)
*Chest tightness*					
Reference, *n* = 211	10 (4.7)	Ref	Ref	Ref	
Exposure only, *n* = 211	16 (7.6)	1.60 (0.73, 3.53)	1.60 (0.66, 3.82)	0.60 (− 0.34, 2.82)	
Current smoking only, *n* = 6	0	NA	NA	NA	
Exposure and current smoking, *n* = 13	2 (15.4)	3.25 (0.71, 14.81)	6.71 (0.95, 47.48)	5.71 (− 0.05, 46.48)	NA
Ex-smoking only, *n* = 11	0	NA	NA	NA	
Exposure and ex-smoking, *n* = 4	0	NA	NA	NA	NA
Reference, *n* = 211	10 (4.7)	Ref	Ref	Ref	
Exposure only, *n* = 211	16 (7.6)	1.60 (0.73, 3.53)	1.60 (0.66, 3.82)	0.60 (− 0.34, 2.82)	
Current or ex-smoking only, *n* = 17	0	NA	NA	NA	
Exposure and Current or ex-smoking, *n* = 17	2 (11.8)	2.48 (0.54, 11.33)	5.25 (0.76, 35.91)	4.25 (− 0.24, 34.91)	NA

Bolded effect estimates are statistically significant at *p* < 0.05

^a^Adjusted for age, marital status, education, BMI, sex, and waterpipe

**Table 5:** Interaction between smoking and occupational exposure among health care workers on lung function parameters

Parameter	Factors	Main effects model β (95% CI)	Interaction model^a^ β (95% CI)	*p*-value
FEV_1_, L	Exposure	− 0.020 (− 0.09, 0.05)	− 0.02 (− 0.09, 0.05)	
	Former smoking	**− 0.25 (− 0.45, − 0.04)**	− 0.24 (− 0.48, 0.004)	
	Current smoking	0.12 (− 0.05, 0.30)	0.11 (− 0.19, 0.42)	
	Exposure × former smoking	NA	− 0.03 (− 0.46, 0.40)	0.93
	Exposure × current smoking	NA	0.02 (− 0.34, 0.37)	0.89
FVC, L	Exposure	− 0.03 (− 0.11, 0.05)	− 0.05 (− 0.13, 0.03)	
	Former smoking	**− 0.26 (− 0.50, − 0.02)**	**− 0.35 (− 0.63, − 0.06)**	
	Current smoking	0.09 (− 0.11, 0.30)	− 0.11 (− 0.46, 0.24)	
	Exposure × former smoking	NA	0.25 (− 0.24, 0.75)	0.32
	Exposure × current smoking	NA	0.28 (− 0.13, 0.70)	0.17
FEV_1_: FVC, %	Exposure	0.06 (− 0.89, 1.01)	0.417 (− 0.56, 1.39)	
	Former smoking	− 0.50 (− 3.32, 2.31)	1.39 (− 1.94, 4.71)	
	Current smoking	1.49 (− 0.96, 3.93)	**5.31 (1.16, 9.46)**	
	Exposure × former smoking	NA	− 5.79 (− 11.66, 0.07)	0.05
	Exposure × current smoking	NA	**− 5.37 (− 10.27, − 0.47)**	0.03
FEV_1_%-predicted	Exposure	− 0.80 (− 2.95, 1.35)	− 0.85 (− 3.08, 1.37)	
	Former smoking	− 6.10 (− 12.28, 0.09)	− 6.21 (− 13.63, 1.21)	
	Current smoking	4.29 (− 1.05, 9.62)	3.23 (− 6.14, 12.60)	
	Exposure × former smoking	NA	0.25 (− 13.07, 13.56)	0.97
	Exposure × current smoking	NA	1.53 (− 9.63, 12.69)	0.78
FVC %-predicted	Exposure	− 0.88 (− 3.04, 1.28)	− 1.26 (− 3.49, 0.97)	
	Former smoking	**− 7.24 (− 13.46, − 1.02)**	**− 9.18 (− 16.62, − 1.73)**	
	Current smoking	1.41 (− 3.95, 6.77)	− 3.181 (− 12.57, 6.21)	
	Exposure × former smoking	NA	5.82 (− 7.53, 19.16)	0.39
	Exposure × current smoking	NA	6.48 (− 4.71, 17.67)	0.25
FEV_1_: FVC %-predicted	Exposure	0.02 (− 1.14, 1.17)	0.39 (− 0.80, 1.58)	
	Former smoking	1.15 (− 2.17, 4.48)	3.20 (− 0.75, 7.16)	
	Current smoking	3.22 (0.35, 6.09)	**7.25 (2.24, 12.25)**	
	Exposure × former smoking	NA	− 6.25 (− 13.36, 0.85)	0.08
	Exposure × current smoking	NA	− 5.64 (− 11.60, 0.32)	0.06

Bolded effect estimates are statistically significant at *p* < 0.05

^a^Adjusted for age (for the actual values), height (for the actual values), sex (for the actual values), marital status, education and waterpipe

Table 5 presents the effect estimates from the linear regression models, both from the main effects models and models with interaction terms included. The interaction for occupational exposure and current smoking was significant on FEV1/FVC (β = − 5.31, 95% CI − 9.46, − 1.16, *p* = 0.03). Marginally significant evidence of interaction between occupational exposure and former smoking was also observed on FEV1/FVC (*p* = 0.05).

## Discussion

We studied the prevalence of respiratory symptoms and level of lung function among staff working in a large and busy hospital in Iran. We found that hospital workers had significantly higher prevalences of cough, phlegm production, productive cough, and wheezing compared to the reference group of office workers from the same hospital but working in the neighbor building. In addition, among the specific healthcare worker groups, nurses, aid nurses and laboratory workers had increased prevalences of several respiratory symptoms. We also observed significant reductions in the levels of FEV_1_ and FVC among the subgroups of nurses and other hospital workers in unadjusted models (Additional file 1: Table S1). Furthermore, the prevalence of restrictive lung function impairment was higher in the exposed group compared to the reference group.

Healthcare workers are exposed to multiple agents potentially harmful for respiratory health. Disinfection of medical instruments in healthcare is likely to expose workers to substances leading to airway inflammation, especially in work tasks requiring high volumes or concentrations of disinfectants. Use of disinfectants to clean surfaces may also be linked to workers’ exposure to chemical agents that may cause adverse respiratory effects. In our study, local exhaust ventilation was used only in a few work areas.

We also addressed potential interaction between current or former smoking and occupational exposures, i.e., whether smoking modifies effects of such exposures. In our study, current smoking was found to have an independent adverse effect on FEV1/FVC%. Lack of any significant effect on the other lung function parameters could be due to the so-called healthy smoker selection, which means that healthier people are more likely to start smoking and to continue it [18]. In addition, the relatively young average age of this study population (mean 36.3 years, SD 8.25) could explain the rather modest effects on lung function, as the subjects had relatively short duration of smoking. In the main effect models, lung function, apart from FEV1/FVC %-predicted, was statistically significantly reduced in former smokers, suggesting lack of a recovery from the adverse effects of previous smoking. The absence of a parallel finding in current smokers is likely explained by two plausible general explanations: (1) the healthy smoker effect, meaning that those susceptible to adverse effects of smoking have already quit smoking while those resistant to these continue smoking, and (2) the relatively young study population with on average short duration of smoking. The interaction between current smoking and exposure to healthcare chemicals was significant on FEV1/FVC%, suggesting synergism between these two exposures.

## Validity of the results and limitations

### Study population

We achieved 100% response rate for both the exposed and reference groups (i.e., after using an incentive), which practically eliminates potential bias that could be related to reduced participation. We compared hospital staff to a reference group of office workers and these two groups were found to have similar demographic and personal characteristics. There were no significant differences with respect to the lifetime smoking between the two groups. The small sample sizes, particularly after including smoking status in the models, generated wide confidence intervals. Although the sample sizes were designed to detect significant effects, they may not be large enough to detect statistically significant interaction.

### Study design

This was a cross-sectional study; therefore, it is not possible to elaborate the possibility that workers with respiratory health problems may have been more likely to have left the work compared with workers who remain healthy [19]. This type of selection would lead to underestimation of the relations of interest for current exposures.

### Outcome and exposure assessment

Occurrence of respiratory symptoms was assessed with the same questionnaire in a similar way among the occupational subgroups of the hospital staff and that of the control group of office workers. In addition, the spirometry measurements were conducted according to same protocol for both the healthcare worker and the office worker groups.

Exposure was assessed in this study with two methods: (1) on the basis of the broad job category, i.e. healthcare worker vs. office worker; and (2) on the basis of subcategory based on job titles. Both types of exposed categories were consistently related to respiratory symptoms. Unfortunately, we were not able to directly measure the occupational exposures and the study size was not optimal for addressing specific exposures.

### Confounding

We collected information on several potential determinants of the studied outcomes, which were adjusted for as potential confounders in the multivariate models: personal characteristics (sex, age), socioeconomic status (education, and marital status), and smoking habits. There is evidence that long-term exposure to air pollution reduces lung function and increases occurrence of respiratory symptoms. We recruited participants of both groups from the same hospital area located in Shiraz, Iran, and thus minimized potential confounding role of air pollution exposure.

### Synthesis with previous knowledge

The most commonly reported exposures among hospital staff in the United States were cleaning products, latex, and poor indoor air quality in general [20]. Some recent studies have shed additional light on the role of cleaning products in hospital environment [9, 10, 21]. In the present study, prevalence of phlegm production was significantly greater in nurses compared to the office workers (i.e. the reference group), which is consistent with a study by Smedbold et al. [22] from Norway. The authors concluded that poor indoor environment might have affected the nasal mucosa of the nursing personnel causing nasal mucosal production.

The high prevalence of respiratory symptoms among hospital staff in our study is consistent with a previous study from United States [20, 23]. In the present study, the most prevalent symptoms among hospital staff were shortness of breath (31.1%) and cough (23.7%). These prevalences are somewhat higher than reported in other studies conducted in different parts of the world. In our study, poor indoor air quality in general and exposure to detergents and disinfectants [24] are possible explanations underlying the higher prevalence of respiratory symptoms in the hospital staff. In our study, the clinical laboratory workers reported the highest prevalence of respiratory symptoms among the hospital group. This finding is consistent with results of the study by Mirabelli et al. [25]. Increased occurrence of symptoms among nurses was also reported in previous studies by Arif et al. [24] and Pechter et al. [20].

The previous studies have adjusted for smoking but have not explored potential modifying effect by it among healthcare workers. We did not identify any previous study that had investigated potential interaction between occupational exposures and smoking among healthcare workers. In our study, the interaction between current smoking and healthcare work exposure was significant in relation to FEV1/FVC, which suggests synergism between these two exposures, i.e., current smokers seemed to be more susceptible to the adverse effects of the exposures in hospitals.

We assume that the environmental conditions and the workers of the studied teaching hospital in Shiraz, Iran, represent well the situation also in other hospitals in Iran and other countries in the same region and in areas with similar environmental and socioeconomic conditions. Thus, the results are generalizable to such areas of the world.

## Conclusions

In this study from Iran, the results showed that the prevalence of all respiratory symptoms, except for chest tightness, was higher among hospital staff compared to the reference group of office workers. Laboratory workers were found to be at the highest risk of experiencing respiratory symptoms compared to the comparison group of office workers and to workers in other hospital jobs. No significant differences were found in lung function between the hospital and office workers. There was a suggestion of a synergistic effect between occupational exposures and current smoking on reduced FEV1/FVC%. Our findings are relevant for policymakers to justify implementation of evidence-based measures to reduce occupational exposures in order to prevent respiratory illness in hospital staff.

## Supplementary Information

**Additional file1.** **Table S1**: Unadjusted and adjusted effects of occupational exposure on lung function parameters.

## Data Availability

The datasets used and/or analyzed during the current study are available from the corresponding author on reasonable request.
