# Supplementary material for: Do hospital workers experience a higher risk of respiratory symptoms and loss of lung function?
Source: BMC Pulm Med. 2022 Aug 8;22:303. doi: 10.1186/s12890-022-02098-5 (PMC9358815; doi:10.1186/s12890-022-02098-5)
Supplement: Supplementary file 1 — Additional file1. Table S1: Unadjusted and adjusted effects of occupational exposure on lung function parameters. [file 12890_2022_2098_MOESM1_ESM.docx]

**Table S1.** Unadjusted and adjusted effects of occupational exposure on lung function parameters.

| **Adjusted model 2 ^b^ β (95% CI)** | **Adjusted model 1 ^a^ β (95% CI)** | **Unadjusted model** |  |  | **Parameter** |
| --- | --- | --- | --- | --- | --- |
| -0.008 (-0.07, 0.06) | -0.004 (-0.07, 0.06) | -0.014 (-0.13, 0.11) |  |  | FEV_1_, L |
| -0.016 (-0.09, 0.06) | -0.01 (-0.09, 0.06) | -0.003 (-0.15, 0.14) |  |  | FVC, L |
| 0.12 (-0.81, 1.07) | 0.15 (-0.78, 1.08) | -0.43 (-1.36, 0.50) |  |  | FEV_1_: FVC, % |
| -0.43 (-2.58, 1.71) | -0.32 (-2.45, 1.79) | -0.56 (-2.59, 1.46) |  |  | FEV_1_ %-predicted |
| -0.58 (-2.74, 1.57) | -0.40 (-2.53, 1.73) | -0.41 (-2.46, 1.63) |  |  | FVC %-predicted |
| 0.1 (-1.04, 1.25) | 0.03 (-1.10, 1.64) | -0.24 (-1.32, 0.85) |  |  | FEV_1_: FVC %-predicted |
|  |  |  | n | Category | FEV_1_, L |
| Ref | Ref | Ref | 228 | Reference Office |  |
| -0.02 (-0.11, 0.07) | -0.02 (-0.12, 0.07) | **-0.20 (-0.36, -0.03)** | 86 | Nurses |  |
| -0.08 (-0.25, 0.08) | -0.06 (-0.23, 0.10) | -0.06 (-0.36, 0.23) | 20 | Laboratory workers |  |
| 0.00 (-0.13, 0.13) | -0.003 (-0.14, 0.13) | 0.05(-0.17, 0.27) | 37 | Nurses’ aides |  |
| -0.01 (-0.17, 0.15) | 0.002 (-0.16, 0.16) | -0.01(-0.25, 0.22) | 33 | Cleaners |  |
| -0.02 (-0.19, 0.14) | -0.01 (-0.19, 0.15) | 0.20 (-0.10, 0.51) | 19 | Surgical technicians |  |
| 0.08 (-0.05, 0.22) | 0.10 (-0.03, 0.23) | 0.30 (0.06, 0.54) | 33 | Others |  |
| Ref | Ref | Ref | 228 | Reference Office | FVC, L |
| -0.05 (-0.16, 0.06) | -0.05 (-0.16, 0.05) | **-0.25 (-0.45, -0.06)** | 86 | Nurses |  |
| -0.09 (-0.28, 0.10) | -0.70 (-0.26, 0.12) | -0.029 (-0.38, 0.32) | 20 | Laboratory workers |  |
| 0.01 (-0.14, 0.17) | 0.001 (-0.15, 0.15) | 0.08 (-0.18, 0.35) | 37 | Nurses’ aides |  |
| 0.00 (-0.18, 0.18) | 0.01 (-0.17, 0.20) | 0.04 (-0.24, 0.32) | 33 | Cleaners |  |
| -0.05 (-0.25, 0.14) | -0.05 (-0.24, 0.14) | 0.23(-0.13, 0.59) | 19 | Surgical technicians |  |
| 0.11 (-0.04, 0.27) | 0.12 (-0.03, 0.28) | 0.40 (0.11, 0.68) | 33 | Others |  |
| Ref | Ref | Ref | 228 | Reference Office | FEV_1_: FVC, % |
| 0.50 (-0.81, 1.81) | 0.46 (-0.84, 1.76) | 0.50 (-0.75, 1.76) | 86 | Nurses |  |
| -0.01 (-2.30, 2.27) | -0.008 (-2.27, 2.25) | -1.26(-3.59, 1.06) | 20 | Laboratory workers |  |
| -0.28 (-2.15, 1.58) | -0.12 (-1.96, 1.70) | -0.67 (-2.44, 1.09) | 37 | Nurses’ aides |  |
| -0.42 (-2.63, 1.78) | -0.36 (-2.56, 1.82) | -1.69 (-3.55, 0.16) | 33 | Cleaners |  |
| -0.01 (-1.70, 2.93) | 0.63 (-1.67, 2.93) | 0.05 (-2.33, 2.43) | 19 | Surgical technicians |  |
| -0.28 (-2.27, 1.47) | -0.34 (-2.19, 1.51) | -1.10 (-2.96, 0.74) | 33 | Others |  |
| Ref | Ref | Ref | 228 | Reference Office | FEV_1_ %-predicted |
| -1.01 (-3.98, 1.96) | -1.01 (-3.96, 1.93) | -1.02 (-3.77, 1.73) | 86 | Nurses |  |
| -2.21 (-7.33, 2.91) | -1.83 (-6.94, 3.27) | -1.48 (-6.56, 3.59) | 20 | Laboratory workers |  |
| -0.08 (-4.32, 4.15) | -0.17 (-4.35, 4.00) | -0.45 (-4.31, 3.40) | 37 | Nurses’ aides |  |
| -1.05 (-6.05, 3.95) | -0.49 (-5.45, 4.46) | -1.88 (-5.94, 2.17) | 33 | Cleaners |  |
| 0.07 (-5.15, 5.31) | 0.21 (-5.01, 5.44) | 0.32 (-4.87, 5.52) | 19 | Surgical technicians |  |
| 2.18 (-2.05, 6.42) | 2.43 (-1.79, 6.66) | 1.86 (-2.19, 5.91) | 33 | Others |  |
| Ref | Ref | Ref | 228 | Reference Office | FVC %-predicted |
| -1.18 (-4.17, 1.79) | -1.11 (-4.07, 1.85) | -0.64 (-3.42, 2.13) | 86 | Nurses |  |
| -2.15 (-7.29, 2.98) | -1.62 (-6.76, 3.51) | -1.32 (-6.44, 3.78) | 20 | Laboratory workers |  |
| -0.07 (-4.32, 4.17) | -0.23 (-4.43, 3.97) | -0.86 (-4.74, 3.02) | 37 | Nurses’ aides |  |
| -0.17 (-5.19, 4.84) | 0.58 (-4.40, 5.57) | -0.67 (-4.76, 3.40) | 33 | Cleaners |  |
| -1.19 (-6.44, 4.06) | -1.02 (-6.28, 4.23) | -0.73 (-5.97, 4.50) | 19 | Surgical technicians |  |
| 1.92 (-2.33, 6.17) | 2.22 (-2.02, 6.48) | 1.69 (-2.38, 5.78) | 33 | Others |  |
| Ref | Ref | Ref | 228 | Reference Office | FEV_1_: FVC %-predicted |
| 0.06 (-1.52, 1.65) | -0.01 (-1.59, 1.55) | -0.54 (-2.02, 0.93) | 86 | Nurses |  |
| 0.03 (-2.71, 2.77) | -0.02 (-2.74, 2.70) | -0.075 (-2.79, 2.64) | 20 | Laboratory workers |  |
| -0.008 (-2.27, 2.25) | -0.08 (-2.32, 2.14) | 0.44 (-1.62, 2.51) | 37 | Nurses’ aides |  |
| -0.83 (-3.51, 1.84) | -0.87 (-3.52, 1.77) | -1.29 (-3.46, 0.87) | 33 | Cleaners |  |
| 1.18 (-1.62, 3.98) | 0.88 (-1.90, 3.68) | 0.95 (-1.83, 3.72) | 19 | Surgical technicians |  |
| 0.22 (-2.05, 2.49) | 0.88 (-2.27, 2.24) | 0.07 (-2.10, 2.23) | 33 | Others |  |

^a^ Adjusted for age (for the actual values), height (for the actual values), marital status, education, and sex (for the actual values), ^b^ Adjusted for age (for the actual values), height (for the actual values), marital status, education, sex (for the actual values), smoking (never, ex, and current), and waterpipe (never, ex, and current).
